# Bleomycin-based electrochemotherapy for recurrent oral CD8-positive epitheliotropic T-cell lymphoma in a dog with durable local control and necrotic regional lymph node changes: a case report

**DOI:** 10.3389/fvets.2026.1942467

**Published:** 2026-09-28

**Authors:** Makoto Akiyoshi, Masaharu Hisasue

**Affiliations:** 1 Akiyoshi Animal Clinic, Yamato, Kanagawa, Japan; 2 Laboratory of Small Animal Internal Medicine, School of Veterinary Medicine, Azabu University, Sagamihara, Kanagawa, Japan

**Keywords:** bleomycin, canine, electrochemotherapy, epitheliotropic T-cell lymphoma, immunogenic cell death, oclacitinib, oral lymphoma, T-cell lymphoma

## Abstract

**Background:**

Oral epitheliotropic T-cell lymphoma (ETCL) is an uncommon form of canine cutaneous lymphoma involving the oral mucosa and mucocutaneous junctions. Information regarding optimal treatment strategies and prognosis is limited. Electrochemotherapy (ECT) has demonstrated efficacy against various cutaneous and subcutaneous tumors in dogs and cats; however, reports describing its use for canine oral ETCL are scarce.

**Case description:**

A 13-year-old male Beagle presented with multiple nodular lesions involving the right maxillary gingiva, buccal mucosa, and mandibular gingiva. Histopathology and immunohistochemistry confirmed CD8-positive epitheliotropic T-cell lymphoma, with more than 90% of neoplastic cells expressing CD3 and CD8. Initial treatment with oclacitinib resulted in a reduction in the size and number of the oral lesions; however, disease progression was observed after 149 days. Bleomycin-based electrochemotherapy (BLM-ECT) was subsequently performed using intravenous bleomycin (20 mg/m^2^) followed by electric pulse delivery with a four-needle electrode (1,000 V/cm, eight pulses). Two BLM-ECT sessions were administered on days 205 and 238, resulting in marked reduction in the size and number of the oral lesions and prolonged local disease control. On day 354, enlargement of the left mandibular lymph node was observed. Histopathological examination revealed extensive necrosis that precluded definitive determination of lymphoma involvement. Although residual CD3-positive lymphocytes were identified, neither flow cytometry nor polymerase chain reaction for antigen receptor rearrangement (PARR) confirmed a neoplastic lymphoid population. A third BLM-ECT session was performed on day 555 for recurrent erythematous lesions of the median upper and lower lip mucosa, in which T-cell lymphoma was histologically confirmed. At the latest evaluation on day 608, no grossly visible oral lesions were detected, and there was no clinical evidence of systemic dissemination. The dog remained clinically well.

**Conclusion:**

BLM-ECT provided prolonged local disease control in a dog with recurrent multifocal oral CD8-positive ETCL that had progressed despite oclacitinib treatment. Unusual necrotic changes also developed in regional lymph nodes. The etiology of these changes remained undetermined, and a causal relationship with ECT could not be established. Investigation of pathological and immunological changes following ECT is warranted. BLM-ECT may represent a feasible and repeatable local treatment option for selected dogs with oral ETCL.

## 1 Introduction

Canine lymphoma is one of the most common hematopoietic malignancies and is generally classified into multicentric, alimentary, mediastinal, extranodal, and cutaneous forms according to the primary anatomical site of involvement (1, 2). Systemic chemotherapy remains the standard treatment for most forms of canine lymphoma (2, 3). Epitheliotropic T-cell lymphoma (ETCL) is a subtype of cutaneous lymphoma characterized by infiltration of neoplastic T lymphocytes into epithelial structures (4–6). Although ETCL most commonly affects the skin, lesions may also occur in the oral cavity, including the lips, oral mucosa, mucocutaneous junctions, gingiva, and tongue (4–7).

Canine oral ETCL is uncommon, and information regarding its biological behavior, optimal treatment, and prognosis remains limited. Previous studies have reported a median survival time of approximately 241 days in dogs with multifocal oral ETCL involving the mucosa and mucocutaneous junctions (7). Chemotherapy and radiation therapy have been described as treatment options; however, no standard therapeutic protocol has been established.

Electrochemotherapy (ECT) combines administration of cytotoxic drugs with the application of short, high-voltage electric pulses to transiently increase cell membrane permeability (8–10). This technique enhances intracellular drug uptake and significantly potentiates the antitumor activity of bleomycin (8–10). ECT has demonstrated efficacy in various canine and feline tumors, including mast cell tumors, soft tissue sarcomas, squamous cell carcinomas, and melanomas (8–10). Although ECT has previously been evaluated for localized lymphoma in companion animals, its use specifically for multifocal canine oral CD8-positive ETCL has not been well characterized (11). The present report describes prolonged local disease control achieved with repeated Bleomycin-based electrochemotherapy (BLM-ECT) in a dog whose oral lesions progressed despite oclacitinib treatment. It also documents unusual necrotic changes in regional lymph nodes during follow-up, the biological significance of which remained uncertain.

## 2 Case description

### 2.1 Signalment and clinical findings

A 13-year-old intact male Beagle weighing 11 kg was referred to our hospital for evaluation of multiple oral masses. According to the owner, oral lesions had been present for approximately 18 months but had increased in both size and number during the preceding month.

Physical examination revealed multiple nodular lesions involving the right maxillary gingiva, right buccal mucosa, and right mandibular gingiva (Figure 1). No enlargement of peripheral lymph nodes was detected. Cytological examination of regional lymph nodes suggested reactive hyperplasia. Complete blood count, serum biochemical analysis, thoracic radiography, and abdominal ultrasonography revealed no clinically significant abnormalities.

**Figure 1:**
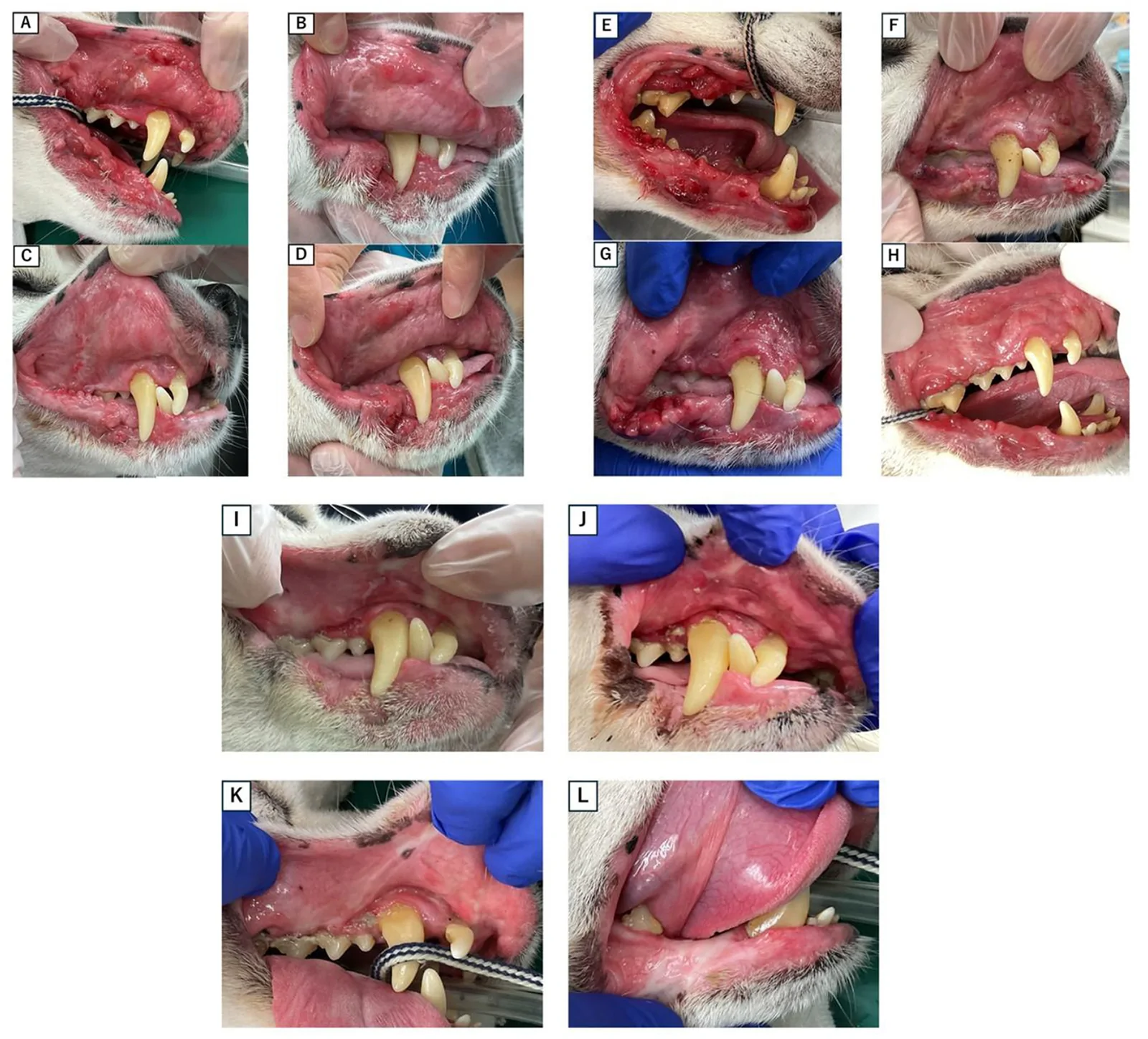
Serial gross appearance of the oral lesions during oclacitinib treatment **(A–D)**. **(A)** Oral lesions at the time of diagnostic biopsy on day 8. **(B)** Visible reduction in the size and number of oral lesions on day 22 after 14 days of oclacitinib administration (0.73 mg/kg, BID). **(C)** Sustained reduction in oral lesion burden on day 120 after reduction of oclacitinib to 0.73 mg/kg, SID. **(D)** Subsequent increase in the size and number of erythematous nodular lesions involving the upper and lower lip mucosa on day 149. Serial gross appearance of the oral lesions during the first and second bleomycin-based electrochemotherapy (BLM-ECT) treatments **(E–H)**. **(E)** Oral lesions immediately before the first BLM-ECT session on day 205. **(F)** Marked visible reduction in the size and extent of the oral lesions 7 days after the first BLM-ECT session (day 212). **(G)** Further reduction of the lesions 21 days after the first BLM-ECT session (day 226). **(H)** Residual oral lesions immediately before the second BLM-ECT session on day 238. Serial gross appearance of the oral lesions following the second BLM-ECT treatment **(I–L)**. **(I)** Persistent reduction in oral lesion burden on day 302, 56 days after the second BLM-ECT session. **(J)** Further reduction of the lesions on day 344, 98 days after the second BLM-ECT session. **(K)** Right upper lip mucosal lesion on day 354, when enlargement of the left mandibular lymph node was detected; no gross progression of the oral lesion was observed. **(L)** Right lower lip mucosal lesion on day 354, likewise showing no gross progression.

### 2.2 Diagnostic assessment

Fine-needle aspiration cytology of the oral lesions was performed without sedation, general anesthesia, or regional analgesia and was suggestive of lymphoma. Histopathological examination of biopsy specimens obtained from the maxillary gingiva, mandibular gingiva, and buccal mucosa revealed sheet-like infiltration of medium-sized lymphoid round cells at the epithelial-subepithelial junction and within the submucosa (Figure 2). The cells had round nuclei with finely stippled chromatin, small single nucleoli, and a small to moderate amount of eosinophilic cytoplasm. Multifocal infiltration of these lymphoid cells into the epithelium, consistent with epitheliotropism, was observed. Mitotic figures were observed at up to 2 per high-power field (0.237 mm^2^).

**Figure 2:**
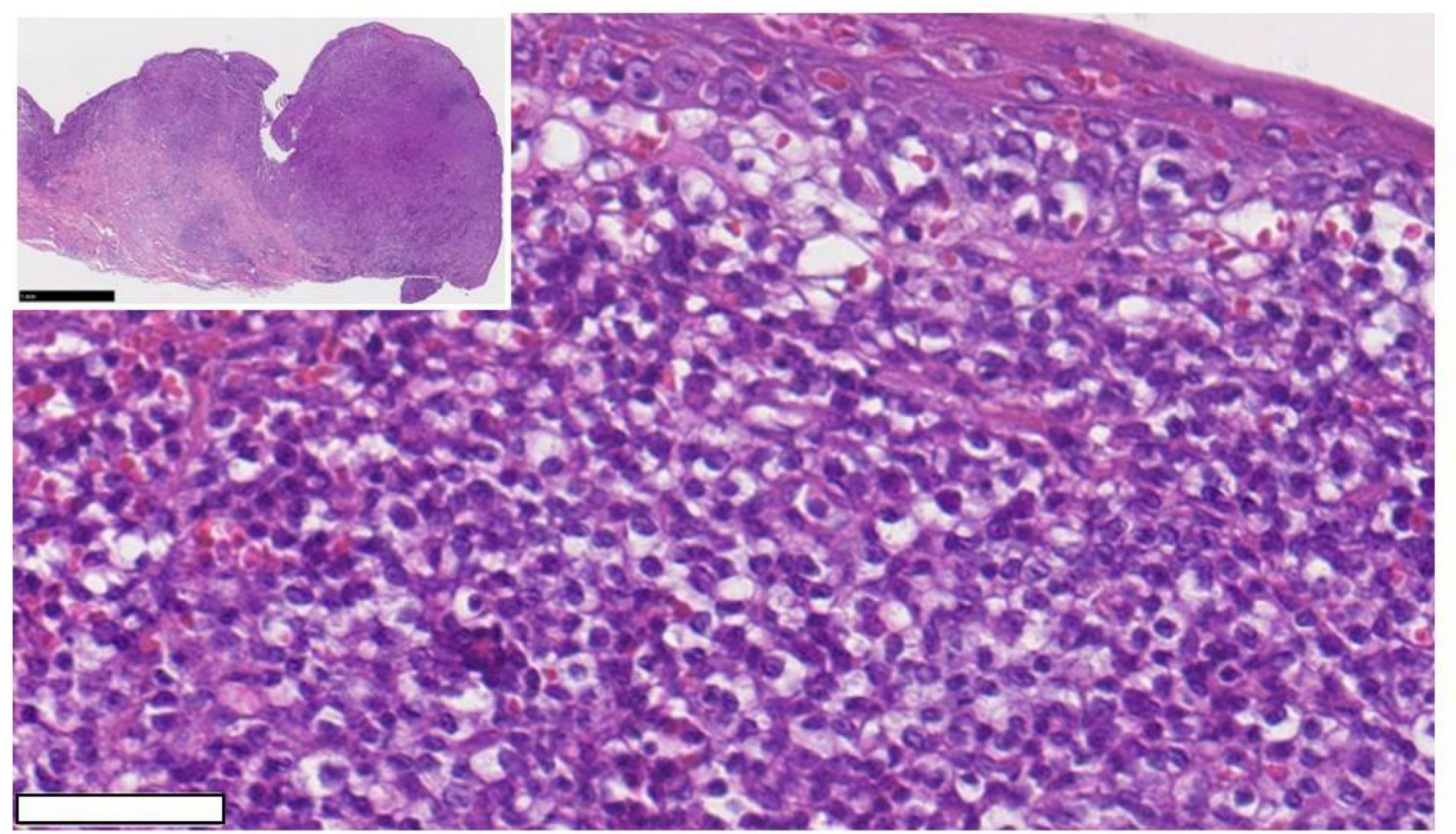
Histopathological findings of the upper lip mucosal lesion. Hematoxylin and eosin (HE) stain demonstrating infiltration and proliferation of medium-sized neoplastic lymphocytes within the epithelium and subepithelial tissues, consistent with epitheliotropic T-cell lymphoma. Scale bar = 50 μm; inset scale bar = 1 mm.

Immunohistochemically, more than 90% of the proliferating lymphoid cells were negative for CD20 and positive for CD3. CD3 immunohistochemistry was performed using a rat anti-human CD3 monoclonal antibody (clone CD3-12; Bio-Rad), which is cross-reactive with canine CD3. For CD8α immunohistochemistry, the rat monoclonal antibody clone F3-B2, specifically established and validated for use in canine tissues, was used (12). For both CD3 and CD8α immunostaining, appropriate staining of resident lymphocytes serving as internal positive controls was confirmed within the same tissue sections, while epithelial and stromal components serving as internal negative controls showed no inappropriate immunoreactivity. More than 90% of the proliferating lymphoid cells were positive for CD8α (Figure 3). PARR and flow cytometry did not demonstrate a distinct clonal or neoplastic T-cell population. Based on the histopathological findings, including the morphologically atypical lymphoid population and prominent epitheliotropism, together with the CD3-positive/CD8α-positive immunophenotype, CD8-positive epitheliotropic T-cell lymphoma was diagnosed.

**Figure 3:**
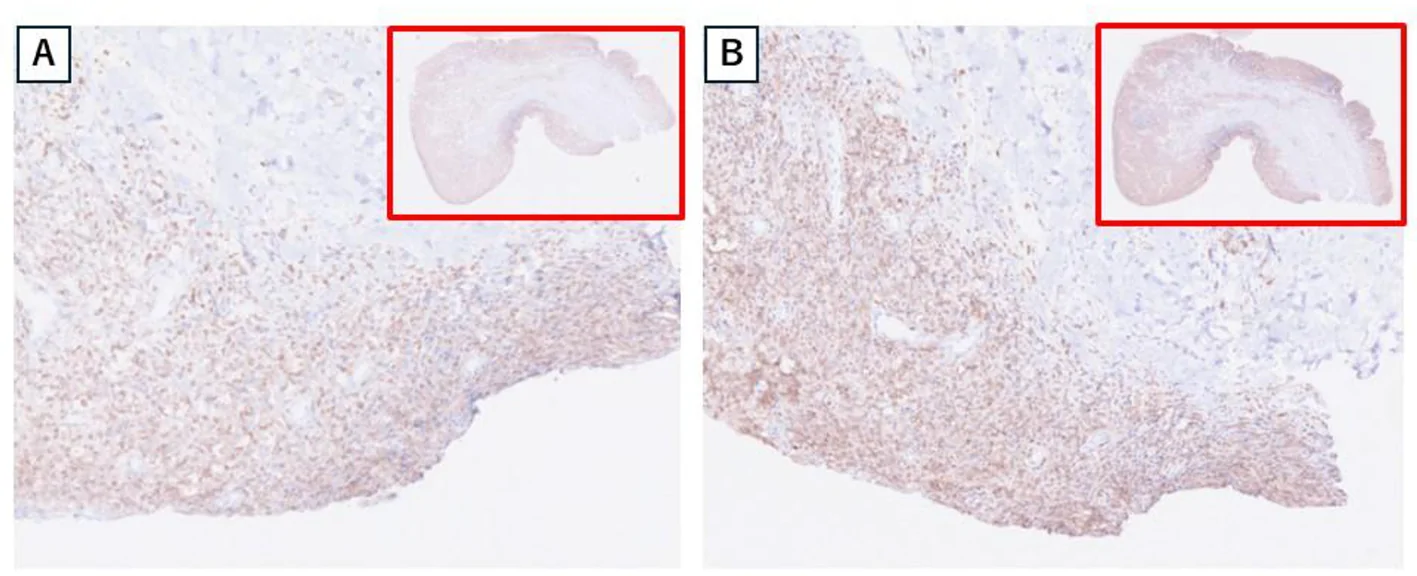
Immunohistochemical findings of the upper lip mucosal lesion. **(A)** CD3 immunostaining. **(B)** CD8α immunostaining using the rat monoclonal antibody clone F3-B2. More than 90% of the neoplastic lymphocytes were positive for both CD3 and CD8α.

### 2.3 Therapeutic intervention

Treatment with oclacitinib [0.73 mg/kg, twice daily (BID)] was initiated on day 8. By day 22, the oral lesions had visibly decreased in both size and number (Figure 1). However, intermittent diarrhea developed, and the oclacitinib dosage was reduced to 0.73 mg/kg, SID.

The oral lesions remained stable for several months; however, recurrence was observed on day 149 (Figure 1). Increasing the oclacitinib dosage back to 0.73 mg/kg, BID failed to control the subsequent increase in the size and number of the oral lesions (Figure 1). Standard treatment options, including systemic chemotherapy [e.g., cyclophosphamide, doxorubicin, vincristine, and prednisone (CHOP)- or CCNU-based protocols] and radiation therapy, were recommended to the owner. However, the owner preferred not to initiate intensive systemic chemotherapy as the first treatment approach and also expressed concerns regarding treatment burden, including the anticipated frequency of hospital visits and financial considerations. Consequently, BLM-ECT was selected as an alternative local treatment strategy after progression during oclacitinib therapy. Administration of a Huaier-derived proteoglycan preparation (Animmune^®^, HACHI Co., Ltd., Japan; TPG-1) as adjunctive therapy was also started.

On day 205, repeat biopsies of the oral lesions were obtained immediately before BLM-ECT. BLM-ECT was performed under general anesthesia induced with propofol (8 mg/kg, IV). A transdermal fentanyl patch (4.2 mg) was applied on the day of ECT for postoperative analgesic support. Bleomycin (Bleo^®^ for Injection; Nippon Kayaku Co., Ltd., Tokyo, Japan) was administered intravenously as a bolus at a dose of 20 mg/m^2^. Eight minutes later, electric pulses were delivered using a veterinary electroporation device (ELECTROvet EZ; LEROY Biotech, France) equipped with a four-needle electrode designed for cavity lesions (Electrode for Cavities; LEROY Biotech, France). The electrode consisted of four needles, each 10 mm in length and 0.68 mm in diameter, arranged in two rows separated by 4 mm. The needles were inserted perpendicular to the mucosal surface so that the visible lesion thickness was encompassed within the treatment field. The manufacturer's preset protocol was used, corresponding to an applied voltage of 400 V and an electric field strength of 1,000 V/cm. Each application consisted of eight square-wave pulses of 100 μs duration delivered at a frequency of 5,000 Hz (8–10). The treatment field included the grossly visible lesions and an approximately 5-mm margin of surrounding mucosa. Electrode placements were sequentially distributed across the irregular lesions and surrounding margins to achieve complete coverage while minimizing unnecessary overlap between adjacent treatment fields. Thirty-five electrode applications were delivered to the right upper lip mucosa and gingiva, and an additional 35 applications were delivered to the right lower lip mucosa and gingiva.

Histopathological examination of the repeat biopsy specimens confirmed findings consistent with the initial diagnosis. Comprehensive staging performed on the same day, including evaluation of peripheral lymph nodes and systemic imaging, revealed no evidence of lymphoma dissemination.

By day 212, a marked visible reduction in the size and extent of the oral lesions was observed (Figure 1). A second BLM-ECT session was performed on day 238 using the same device, electrode, drug dose, and pulse protocol (Figure 1). Twenty-nine electrode applications were delivered to the right upper oral lesions and 25 to the right lower oral lesions. The reduced number of electrode applications during the second session reflected the decreased extent of the grossly visible lesions following the first BLM-ECT treatment. Flow cytometry of the right mandibular lymph node performed around the first and second ECT sessions showed mixed lymphoid populations without a definitive neoplastic T-cell population.

Transient swelling and localized purulent inflammation of the treated oral mucosa, accompanied by mild malodor, developed after the second ECT session but resolved following administration of oral dexamethasone (0.1 mg/kg, BID) and oral amoxicillin (15 mg/kg, BID) for 7 days. No clinically apparent systemic adverse events attributable to bleomycin were observed during treatment or subsequent follow-up. By day 260, the oral lesions showed further visible reduction in size and extent (Figure 1).

On day 354, enlargement of the left mandibular lymph node was detected during a follow-up examination. Fine-needle aspiration was inconclusive, and excisional biopsy was subsequently performed. Histopathological examination revealed extensive necrosis replacing most of the lymph node architecture, thereby limiting reliable morphological assessment (Figure 4). Small residual aggregates of CD3-positive lymphocytes were present; however, these findings were insufficient to confirm lymphomatous involvement. Flow cytometry failed to identify a distinct lymphoid population and predominantly detected CD11b/CD11d-positive cells, while PARR analysis was negative. Consequently, the etiology of the necrotic lymph node lesion remained undetermined. Differential interpretations included lymphoma involvement with extensive necrosis, reactive inflammatory change, and a possible treatment-associated regional response.

**Figure 4:**
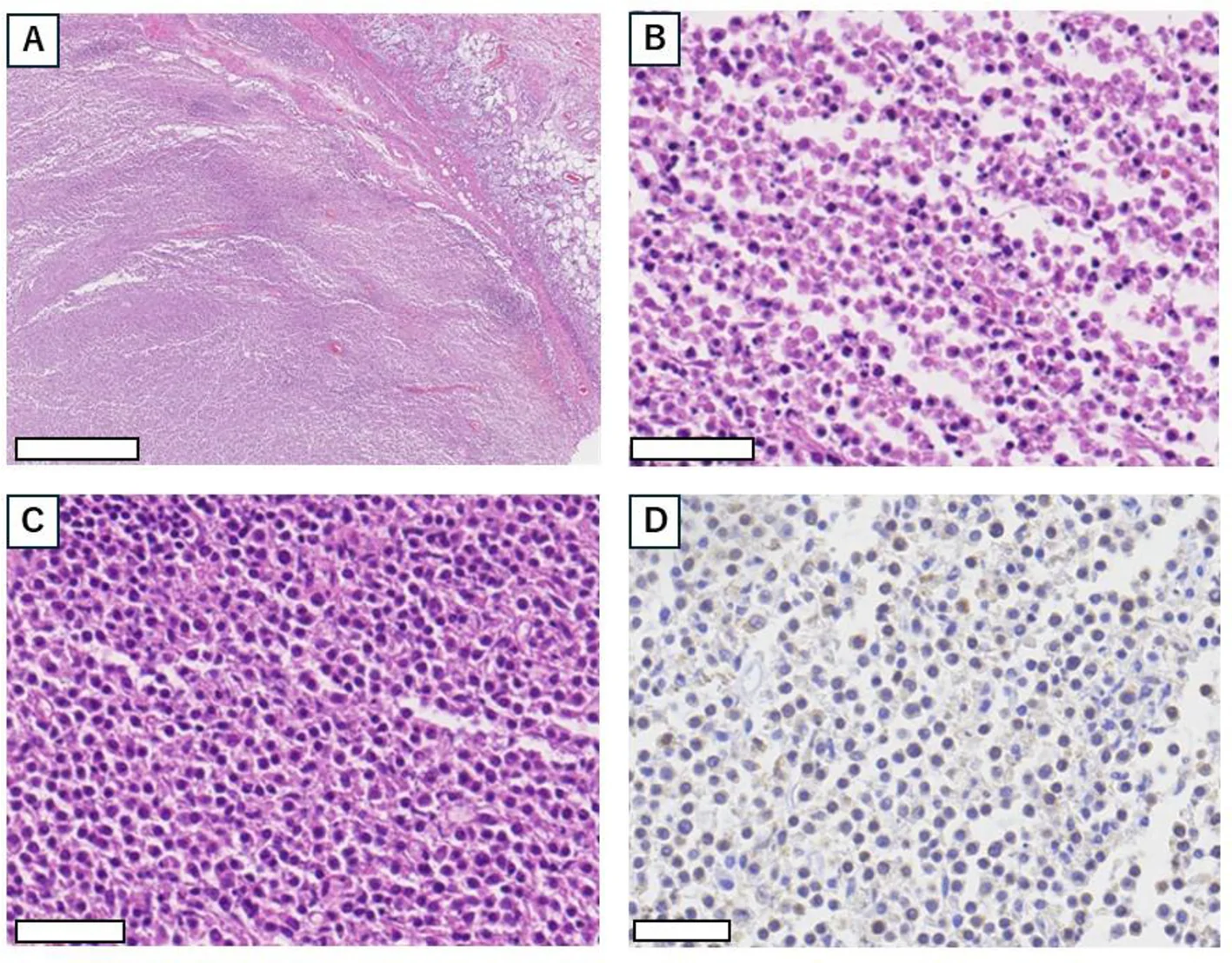
Histopathological findings of the excised left mandibular lymph node. Hematoxylin and eosin (HE) stain. **(A)** Low-power view showing extensive necrosis replacing most of the normal lymph node architecture. Scale bar = 1 mm. **(B)** High-power view of the necrotic area. Scale bar = 50 μm. **(C)** Small residual aggregate of lymphoid cells identified within the excised lymph node. Scale bar = 50 μm. **(D)** CD3 immunostaining. Although the residual lymphoid cells were CD3-positive, extensive necrosis precluded definitive determination of lymphomatous involvement. Scale bar = 50 μm.

On day 555, several recurrent erythematous lesions were identified on the median upper and lower lip mucosa. Biopsy specimens obtained from these lesions again confirmed T-cell lymphoma, and a third BLM-ECT session was performed using the same protocol. On day 573, transient enlargement of the ipsilateral parotid lymph node region was noted. Cytological evaluation showed predominantly necrotic and inflammatory material without definitive evidence of lymphoma. The dog was treated with oral dexamethasone (0.1 mg/kg, SID) and oral amoxicillin (15 mg/kg, BID) for 7 days. The enlarged parotid lymph node completely resolved following treatment, and no recurrence was observed during the subsequent follow-up period. At the most recent evaluation on day 608, no grossly visible oral lesions were detected and there was no clinical evidence of systemic dissemination. The dog remained clinically well.

## 3 Discussion

Canine oral epitheliotropic T-cell lymphoma (ETCL) is an uncommon manifestation of cutaneous lymphoma, and information regarding its optimal treatment and prognosis remains limited (6, 7). Previous studies have reported a median survival time of approximately 241 days in dogs with multifocal oral ETCL involving the oral mucosa and mucocutaneous junctions (6, 7). Lomustine (CCNU) is among the most commonly reported systemic treatments for canine epitheliotropic lymphoma. Retrospective studies have reported overall response rates of approximately 78%−83% with CCNU, although responses were generally of limited duration, with median response durations of approximately 94–106 days (13, 14). Available data specifically for oral ETCL remain limited because most dogs in these studies had cutaneous disease. A retrospective study evaluating primarily radiation-based treatment reported a median survival time of up to 770 days (15). However, direct comparison between studies should be interpreted cautiously because of differences in case selection, disease distribution, treatment protocols, and the inclusion of adjuvant chemotherapy in some patients (15). In the present case, systemic chemotherapy including CCNU was recommended but was not selected because the owner preferred not to initiate intensive cytotoxic chemotherapy as the first treatment approach. BLM-ECT was therefore selected as an alternative local-control strategy rather than because CCNU was considered ineffective.

Oclacitinib is a Janus kinase (JAK) inhibitor that has been reported to exhibit activity against canine cutaneous T-cell lymphoma (16–18). In the present case, the oral lesions initially decreased in size and number following oclacitinib administration. This observation is consistent with previous reports suggesting potential clinical activity of JAK inhibition in canine epitheliotropic lymphoma. However, disease progression occurred despite continued treatment, indicating that the response was temporary. Because this report describes a single case and concurrent biological factors were not investigated, no conclusions regarding the underlying molecular mechanism can be drawn.

Prolonged local disease control was observed following repeated BLM-ECT; however, the contribution of treatment cannot be separated from the potentially indolent biological behavior of the disease in this single case. The oral lesions had reportedly been present for approximately 18 months before presentation, suggesting relatively slow biological behavior before therapeutic intervention. Nevertheless, the lesions showed documented progression despite continued oclacitinib treatment immediately before BLM-ECT and subsequently showed marked reduction following the first two BLM-ECT sessions. Local recurrence on day 555 was treated with a third session, and no grossly visible oral lesions were detected on day 608. Thus, while a causal relationship between BLM-ECT and the prolonged clinical course cannot be established, the temporal association between treatment and local lesion reduction supports further investigation of BLM-ECT as a local treatment strategy for oral ETCL.

Although the histopathological and immunophenotypic findings strongly supported the diagnosis of CD8-positive ETCL, PARR and flow cytometry did not independently demonstrate a clonal or distinct neoplastic T-cell population. Histopathology and immunohistochemistry therefore remained the primary basis for diagnosis in this case. Appropriate internal positive and negative staining patterns were confirmed for the CD3 and CD8α immunohistochemical assays, supporting the specificity of the observed immunoreactivity. However, the lack of additional positive-control confirmation using well-characterized external positive-control tissue represents a limitation and reduces the certainty of the immunohistochemical interpretation. Nevertheless, the absence of independent molecular confirmation and the unusually prolonged clinical course represent limitations in the biological characterization of this lymphoma. A reactive or atypical indolent lymphoid proliferation cannot be completely excluded; however, this possibility was considered less likely because of the morphologically atypical lymphoid population, prominent epitheliotropism, and diffuse CD3/CD8α immunophenotype. Furthermore, previous studies have demonstrated that molecular clonality is not detected in all histologically confirmed cases of canine epitheliotropic T-cell lymphoma (19).

An additional noteworthy finding was the development of an extensively necrotic left mandibular lymph node lesion during follow-up. Histopathology, immunohistochemistry, flow cytometry, and PARR did not permit definitive determination of whether the lymph node had been involved by lymphoma. Residual CD3-positive lymphocytes were present, but extensive necrosis substantially limited morphological interpretation. Flow cytometry did not identify a distinct neoplastic T-cell population, and PARR was negative. Accordingly, lymphoma involvement with extensive necrosis, reactive inflammatory change, and a possible regional treatment-associated response remained differential interpretations. The fact that the primary oral lesions were predominantly right-sided whereas the necrotic lesion occurred in the left mandibular lymph node does not exclude a biological association because crossover lymphatic drainage may occur in the oral cavity (20); nevertheless, the relationship between the lesions remained uncertain. One possible explanation is that the lesion represented regional lymphoma involvement accompanied by extensive spontaneous necrosis. Alternatively, reactive inflammatory changes may have contributed to the observed pathology.

In human medicine, recent evidence suggests that electrochemotherapy may exert immunomodulatory effects in addition to direct cytotoxic activity (21). Electrochemotherapy has been reported to induce immunogenic cell death (ICD), leading to the release of tumor-associated antigens and damage-associated molecular patterns (DAMPs), thereby promoting antitumor immune responses within regional lymphatic tissues (21, 22). In the present case, the occurrence of a predominantly necrotic lymph node lesion after ECT raises the possibility that treatment-associated immunological mechanisms may have contributed to the observed pathological changes. This hypothesis remains speculative and could not be confirmed in the present case. Furthermore, a transient enlargement of the left parotid lymph node region characterized cytologically by necrotic and inflammatory changes was observed on day 573. Histopathological confirmation was not obtained for this lesion; however, it is noteworthy that both lymph node-associated events occurred following ECT treatment.

A Huaier-derived proteoglycan preparation (Animmune^®^, HACHI Co., Ltd., Japan; TPG-1) was administered concurrently during part of the treatment period (23). Its contribution to the clinical course cannot be excluded; however, evidence supporting its efficacy in canine oral ETCL is currently lacking. Therefore, the observed clinical response cannot be attributed exclusively to BLM-ECT.

The present report has several limitations. First, it describes a single case, and no definitive conclusions regarding efficacy can be drawn. Second, the dog received oclacitinib, dexamethasone, and TPG-1 during portions of the follow-up period, and their contribution to disease behavior cannot be excluded. Third, repeated ECT sessions were required; therefore, the duration of response attributable to an individual session could not be determined. Fourth, the irregular and multifocal nature of the oral lesions limited precise objective measurement of tumor burden. Fifth, definitive characterization of the mandibular lymph node lesion was not possible because of extensive necrosis. Finally, standard systemic chemotherapy and radiation therapy were declined, precluding comparison with conventional treatments. Nevertheless, the prolonged local disease management and unusual regional lymph node pathology warrant further investigation. In addition, the unusually prolonged pretreatment history suggests that an intrinsically indolent biological phenotype may have contributed to the clinical course; therefore, prolonged disease control cannot be attributed solely to BLM-ECT.

Overall, the findings of this case suggest that bleomycin-based electrochemotherapy may be a feasible local treatment option for dogs with oral CD8-positive ETCL and support further evaluation of ECT in this rare lymphoma subtype.

## 4 Conclusion

In conclusion, repeated BLM-ECT was associated with prolonged local disease control in a dog with recurrent multifocal oral CD8-positive epitheliotropic T-cell lymphoma that had progressed despite oclacitinib treatment. At the latest evaluation on day 608, no grossly visible oral lesions were detected, and there was no clinical evidence of systemic dissemination. However, the contribution of BLM-ECT cannot be separated from the potentially indolent biological behavior of the disease in this single case, and the absence of independent molecular confirmation warrants caution in attributing the prolonged clinical course primarily to treatment efficacy. Although local recurrence required a third ECT session, the overall clinical course suggests that BLM-ECT may represent a feasible repeatable local treatment option for selected dogs with this uncommon lymphoma subtype. Unusual necrotic changes were also observed in regional lymph nodes; however, their etiology remained undetermined, and a causal relationship with ECT could not be established. Further studies are required to define the indications, efficacy, safety, and optimal retreatment protocols for ECT in canine oral ETCL.

## Data Availability

The raw data supporting the conclusions of this article will be made available by the authors, without undue reservation.
